# Propafenone-Induced QRS Widening in a Child With Arrhythmogenic Right Ventricular Cardiomyopathy: A Case Report and Literatures Review

**DOI:** 10.3389/fped.2020.481330

**Published:** 2020-10-30

**Authors:** Yan-qiu Chu, Ce Wang, Xue-mei Li, Hong Wang

**Affiliations:** Department of Pediatrics, Shengjing Hospital, China Medical University, Shenyang, China

**Keywords:** arrhythmogenic right ventricular cardiomyopathy (ARVC), propafenone, sotalol, QRS widen, sudden cardiac death, implantable cardioverter defibrillator

## Abstract

Arrhythmogenic right ventricular cardiomyopathy (ARVC) is a rare cardiac disease in children, and can lead to sudden cardiac death (SCD). Propafenone is classI_C_ antiarrhythmic medication, and its side effects include cardiovascular compromise in the form of hypotension, bradycardia, ventricular dysrhythmias, QRS widening, and heart block. Propafenone has been reported causing QRS widening, but rarely in children. In this article, we presented a boy diagnosed with ARVC who meets diagnosis criteria based on typical symptoms, electrocardiograph (ECG), echocardiography (Echo), cardiac magnetic resonance imaging (CMRI), sudden death of first family member, and genetic mutation in desmosomal DSG2 gene. Antiarrhythmic drugs have been used for treating patients with ARVC, by eliminating or decreasing the occurring frequency of arrhythmias. As his ECG showed frequent premature ventricular contractions (PVC), he was prescribed with oral propafenone. One day after the drug treatment, he presented dizziness accompanied with significant QRS widening in ECG. His dizziness was improved when Propafenone dose was reduced, and resolved after sotalol replacement, with ECG recovered to nearly normal state of QRS. Propafenone may lead to QRS widening and increase the risk of ventricular tachycardia, and it may not reduce ARVC associated mortality. This report may serve as a precaution for clinicians when providing cares for ARVC patients.

## Background

Arrhythmogenic right ventricular cardiomyopathy (ARVC), also known as “arrhythmogenic right ventricular dysplasia (ARVD),” is characterized by ventricular arrhythmias, right ventricular dysfunction, systolic dysfunction, ventricular dilatation, and fibrofatty replacement of myocardium. It is a progressive disease that may result in heart failures. ARVC patients are at a high risk of sudden cardiac death (SCD) (1). ARVC is a rare autosomal dominant disease. It was first described by Dr. Frank Marcus as a progressive hereditary cardiomyopathy with a higher risk of SCD. The prevalence of ARVC in the general population is about 0.02–0.05% (2). This is one of the main causes of sudden death in young patients due to arrhythmia, and is the leading cause of sudden death in young athletes (3, 4). Genetic variations in desmosomal genes, which are responsible for maintaining intercellular adhesion integrity, have been indicated as pathogenesis in at least 50% of ARVC. Dysfunctional desmosomes cause isolation and death of cardiomyocytes, and thus lead to inflammation in the affected myocytes, followed by apoptosis and replacement of fatty fibrous tissue in heart muscle. Ventricular fibrillation (VF) may occur during cell death, while ventricular tachycardia (VT) is associated with fibro-fatty scars. The typical ECG features are VT with left bundle branch block pattern (LBBB), epsilon (ε) wave and T wave inversion (5–7), potentially leading to SCD in young patients (8). Disease management is focusing on preventing lethal events such as SCD. Implantable cardioverter defibrillator (ICD) has been proven as a beneficial intervention for reducing SCD significantly in ARVC patients. Despite the lack of evidence in reducing SCD, antiarrhythmic drugs like beta blockers and others have been used in treating patients in USA and the Netherlands. Although they don't reduce SCD, but they manage arrhythmias in these patients (9). However, usage of Antiarrhythmic drugs may increase the risk of arrhythmia in ARVC patients with structural cardiomyopathy; these patients have poor tolerance to severe arrhythmias such as ventricular tachycardia. Antiarrhythmic drugs may cause QRS widening, and QRS widening leads to abnormal electrical activities accompanied by abnormal depolarization and repolarization that provide the basis for the formation of fatal ventricular arrhythmias. Widened QRS has been reported to increase the risk of heart failure and sudden death (10, 11). Therefore, clinicians should take precautions when choosing antiarrhythmic drugs for treatment in ARVC patients.

## Case Presentation

A 10-year-old previous health boy was presented with intermittent dyspnea and chest pain over one month without syncope. His older brother was diagnosed with cardiomyopathy in 2001, characterized with enlarged right ventricle and perforated RV, and suddenly died at 16 years old after running in 2007. His mother occasionally had chest tightness. Her ECG reveals T wave inversion in leads of III, V_1_,V_2_, and V_3_, and her Echo revealed a slight decrease of diastolic function, other measurements are in normal range.

**Figure 1:**
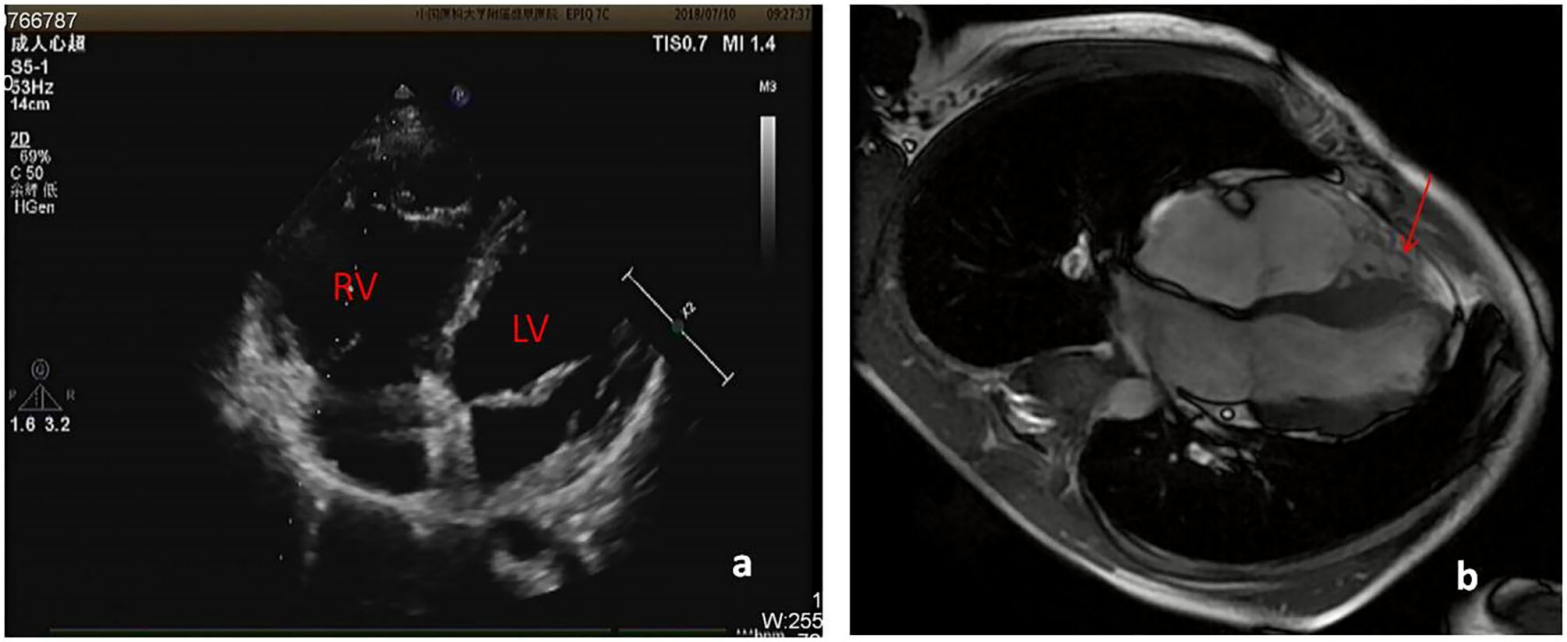
**(a)** Echo showed RV diameter 31.8 mm, the wall of the RV became thinner. **(b)** CMRI showed foci of fat in right ventricular free wall.

**Figure 2:**
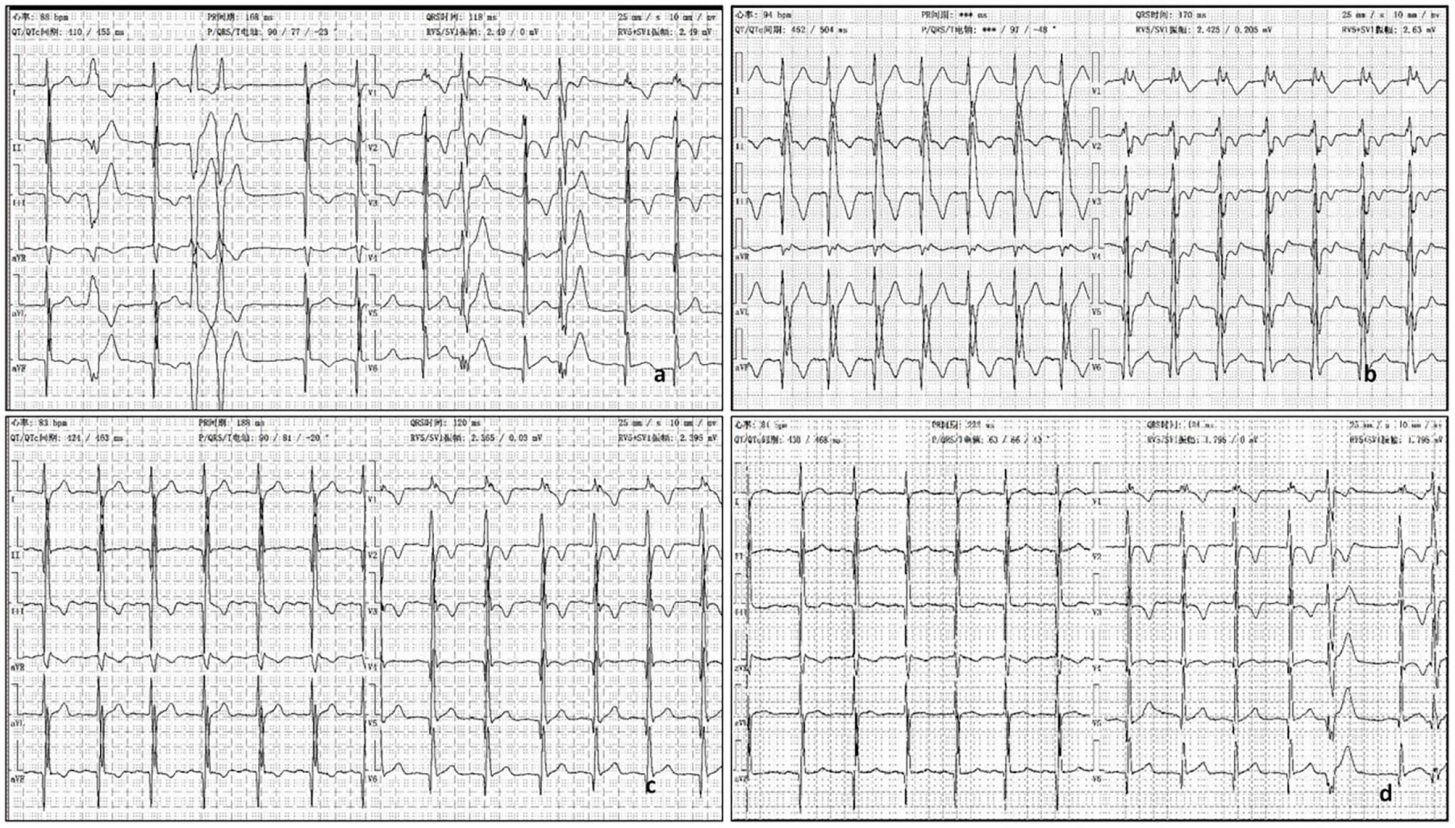
**(a)** ECG showed ε wave, paired PVC, QRS = 118 ms. **(b)** The third day after oral propafenone 200 mg, q8h, ECG showed QRS =170 ms, with dizziness. **(c)** The second day after half oral propafenone 100 mg, q8h, ECG showed QRS = 120 ms. **(d)** After 6 month oral sotalol 80 mg, q12h, ECG showed QRS = 124 ms.

About one month ago, he felt labored dyspnea, and 1 week later he was diagnosed with myocarditis in a local hospital and treated with digoxin, diuretic and myocardium nutrition. Then his symptom released. One week after that, he had dyspnea again and 10 days later was admitted in our pediatric cardiology department. Physical examination showed he was in a general state, Heart Rate 88 bpm, Respiratory Rate 19 bpm, BP 112/72 mmHg, weight 44 kg. No edema on the face and legs, heart sounds strong, without murmur, but rhythm was irregular, auscultation revealed 5 premature beats in 1 min. The liver was 3 cm below right costal margin. Admission blood test revealed normal cTnI, hs-cTnT, CK-MB mass, and NT-pro BNP. Echo showed the right ventricular (RV) was significantly dilated (RV diameter 31.8 mm), RVED/LVED = 0.67, the wall of the RV became thinner, interwoven reticular muscle fiber structure can be seen in the RV (Figure 1). ECG showed ε wave and paired PVC (Figure 2a). Holter showed paired PVC and premature atrial contractions (PAC). Cardiac magnetic resonance imaging (CMRI-in local hospital) revealed a dilated right ventricular outflow tract, thin right ventricular wall with reduced wall motion, and foci of fatty deposit in the right ventricular free wall (Figure 1). Genetic testing finds DSG2 mutation (R49H), which has been indicated to be associated with ARVC. The patient was diagnosed of ARVC.

His father rejected the prescription of betaloc as a treatment for its side effect in reducing sexual function. The patient was treated with aspirin (50 mg, qd po), propafenone (200 mg, q8h), enalapril (5 mg qd), furosemide (20 mg qd, oral 4 days a week), and spironolactone (20 mg qd, oral 3 days a week). At admission, his ECG showed QRS was nearly normal, 118 ms (Figure 2a). QRS duration in this article is measured with V_5_ andII leads. But on the second day after taking oral propafenone, he developed dizziness and his ECG showed QRS was 158 ms. On third day of oral propafenone, QRS was increased to 170 ms (Figure 2b). We reduced the dose of propafenone by half, and on next day, QRS was reduced to 120 ms (Figure 2c). After 1 month treatment, his ECG showed QRS was 164 ms. Therefore, we discontinued propafenone and replaced with sotalol (80 mg, q12 h), and gradually increased the dose to 120 mg. q12 h. At 3 month of illness, QRS was 114 ms; AECG showed PVC 3001/d with paired PVC. Sotalol was then reduced to 80 mg q12 h. At 6 months of illness, QRS returned to normal—ECG showed QRS was 124 ms (Figure 2d). The boy felt better, and the symptoms of dyspnea and chest pain were released.

## Discussion

ARVC is one of the common cause of sudden cardiac death in youth (3, 4). Therefore, young patients with unexplained syncope, cardiac arrest or arrhythmia should be aware of this disease. Patients can present with a wide spectrum of symptoms. Some patients can be asymptomatic, some patients may have palpitations, dizziness, syncope, heart failure, ventricular arrhythmia, or cardiac arrest. Therefore, the diagnosis of ARVC is relatively challenging. We summarize some of the features of ARVC to help us identify this disease early. i Arrhythmias: usually with LBBB or VT, may also present with frequent PVC. In some patients, ECG can find the electrocardiographic epsilon (ε) wave. The ε wave is a low-amplitude spike between the QRS complex and initial portion of the ST segment, and is usually closed to the end of the QRS complex. It is a characteristic ECG waveform in ARVC, and helps clinicians identify possible ARVC earlier (7). ii SCD: The incidence of SCD in ARVC patients under 65 years is ~5% (12). A possible cause of SCD is due to VT developed into ventricular fibrillation (VF). iii Heart Failure: expansion, thinning of RV may lead to right heart failure, and possible left heart failure (13). In this case, the ε wave helps us consider ARVC before the final confirmative diagnosis.

**Figure 3:**
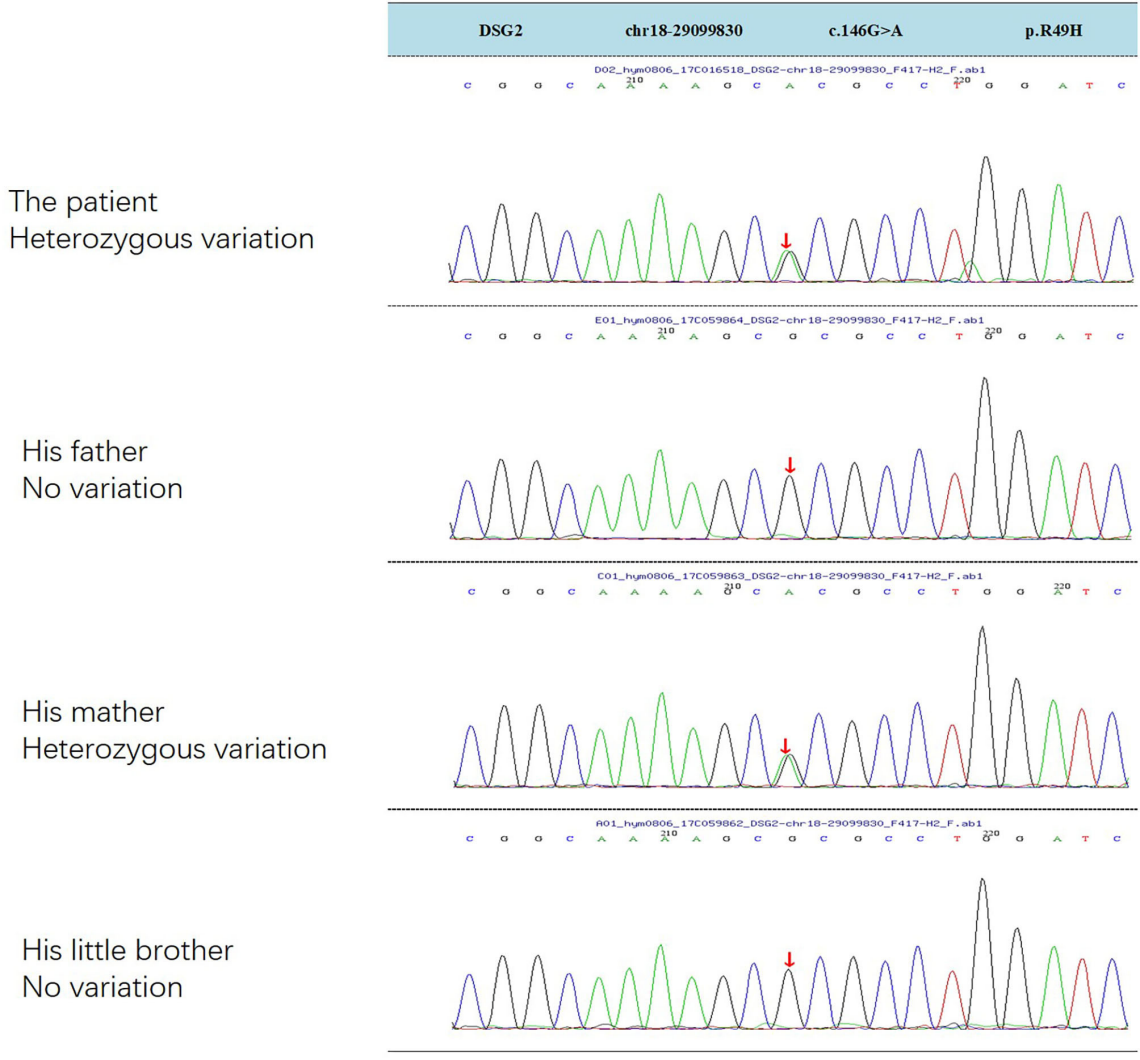
DSG2 gene heterozygous mutation was inherited from his mother.

ARVC is a rare hereditary disease, and 50% of ARVC are caused by autosomal dominant genetic mutations, mainly in desmosomal genes, including plakophilin-2(PKP2), plakoglobin (JUP), desmoglein-2(DSG2), desmocollin-2 (DSC2), and desmoplakin (DSP) (14–16). DSG2 mutation has been suggested as a significant pathogenic mutation causing VT and SCD (17). In this case, genetic testing reveals a heterozygous mutation in DSG2 gene, NM_001943.4:c.146G>A/p. (R49H), which is inherited from his mother (Figure 3). Recent study finds that compound DSG2 mutation variants account for 25% in the largest Chinese ARVC cohort (18). Chen et al. had reported one pedigree with ARVC linked with DSG2 gene complex heterozygous mutation NM_001943.4:c.146G>A/p. (R49H) (the same mutation in this boy), and this mutation is considered as a contributing factor to the severe phenotype in ARVC patients (19). One ARVC diagnosis criteria depends on myocardial biopsy showing a fibrofatty replacement of cardiomyocytes, but the procedure is difficult to perform. In 1994, the Task Force reported a diagnostic Criterion based on major and minor criteria including structural, genetic, ECG, arrhythmia and histological factors (20). Modifications were made in 2002, but it was still less sensitive for patients at the early stage (21, 22). In 2010, this Criterion was further revised in the Task Force recommendations. New task force criteria use histological, genetic, electrocardiographic, and imaging parameters to classify patients into definite, borderline, and possible diagnostic categories (1). But current clinical trial indicates that many pediatric patients do not meet the current ARVC/D diagnostic criteria, and probably are delayed in diagnosis and treatment (23).

Evidence from clinical trials has not proven antiarrhythmic drugs can reduce SCD. However, antiarrhythmic drug could be theoretically beneficial for ARVC patients by eliminating or decreasing frequent occurrence of arrhythmias (9). Beta-blockers, particularly sotalol, are conventionally recommended as the first approach in patients with frequent PVC or non-sustained ventricular tachycardia (24). However, a recent report suggests that neither beta-blocker nor sotalol seem to reduce ventricular arrhythmia (25). Therefore, sotalol has been questioned to be used for treating ARVC. In 2015, European Society of Cardiology proposed that beta-blocker titrated to the maximally tolerated dose could be used as the first-line therapy to improve symptoms in patients with frequent PVC and non-sustained ventricular tachycardia (26). We recommended betaloc for this patient. But his father refused to allow the treatment after being informed potential side effects in sexual function. We choose propafenone after thorough consideration. Propafenone is class I_C_ antiarrhythmic medication that has a structure similar to β-blocker. It blocks the fast-inward sodium current at 0 phase during action potential depolarization, thus causes QRS widening. It slows down the rate of action potential. This prolongs the conduction in myocardium and increases repolarization time, including a slight effect on intraventricular conduction. We input key words “propafenone with wide QRS” when searching in PubMed, there were 5 cases reported in recent 20 years, listed in Table 1. However, it is rarely seen in children with normal cardiac structure that propafenone causes QRS widening. In this case the widening effect is significant. We replaced it with sotalol, and widened QRS returned to normal gradually. Antiarrhythmic drugs may increase the risk of arrhythmia in cardiomyopathy patients with abnormal cardiac structure. These patients have poor tolerance to severe arrhythmias such as ventricular tachycardia. Antiarrhythmic drugs may cause QRS widening, and QRS widening leads to abnormal electrical activities accompanied by abnormal depolarization and repolarization that provide the basis for the formation of fatal ventricular arrhythmias. Widened QRS has been reported to increase the risk of heart failure and sudden death (10, 11). Therefore, clinicians should take precaution when choosing antiarrhythmic drugs in these patients. In ARVC patients who meet the criteria, the right ventricle muscle is gradually replaced with adipose and fibrous tissue, which often leads to ventricular dilatation. Once QRS widening occurs, we should pay great attention as it can easily cause ventricular tachycardia in those patients.

**Table 1:** Reported cases of propafenone induced QRS widening.

**References**	**Age/sex**	**Primary disease**	**Propafenone dose**	**Combination medication**	**Symptoms**	**ECG**
Mantovan et al. (27)	53 y/F	Tachycardia	1 mg/kg iv			Wide QRS
Brubacher et al. (28)	73 y/F	Atrial fibrillation	300 mg/day po	Propranolol	Dyspneic and collapsed, unconscious, no obtainable blood pressure	Wide complex tachycardia
Yeung et al. (29)	72 y/F	Paroxysmal atrial fibrillation	2*300 mg/day po	Verapamil	Vomiting and diarrhea, central chest discomfort, hypotensive, bradycardic	Atrial fibrillation, a QRS duration of 166ms and a corrected QT interval of 536ms
Bayram et al. (30)	25 y/M	Atrial fibrillation	450 mg/day po		Lethargic and blood pressure was not measurable	Widened QRS interval of 210ms, bradycardia with first-degree atrioventricular block
Tomcsányi et al. (31)	84 y/F	Supraventricular tachyarrhythmia	3*150 mg/day po		Severe, continuous palpitation, and a blood pressure of 80/60 mmhg.	Tachycardia with bizarre, wide QRS complexes

*M, male; F, female*.

At present, prevention of the occurrence of fatal events is the main goal in ARVC patient management, and this includes ICD (32). Currently, ICD placement is an effective intervention to reduce mortality in ARVC patients. Other treatment managements including antiarrhythmic drugs, radiofrequency ablation (RFA), cardiac transplantation, and lifestyle changes have not shown significant benefits in reducing mortality (33–35). ICD placement has been used as primary or secondary prevention for ARVC patients (36, 37). For patients with LV dysfunction or severe RV systolic dysfunction, with a family history of sudden death, or with syncope but not excluding possibilities caused by VT or VF, and for patients with higher expectation of survival, ICD placement can be considered as the primary intervention (38).

## Conclusion

Propafenone may lead to QRS widening and increase the risk of ventricular tachycardia occurrence. This may be one of the reasons that propafenone does not reduce the mortality in ARVC patients.

## Ethics Statement

We have obtained written consent from the patient's parents in this case report.

## Author Contributions

Y-qC and HW: conception or design of the work. CW and X-mL: case collection. Y-qC and HW: case analysis and interpretation. Y-qC: drafting the article. HW: critical revision of the article. All authors contributed to the article and approved the submitted version.

## Conflict of Interest

The authors declare that the research was conducted in the absence of any commercial or financial relationships that could be construed as a potential conflict of interest.
